# Milk

**Published:** 1922-11

**Authors:** 


### MILK.

### (1) THE ADMINISTRATION OF THE LAW RELATING TO THE SALE OF MILK.

### (2) PURE AND CLEAN MILK.

THESE two aspects of the milk question were the subject of two very interesting papers read at the Sanitary Inspectors' Conference at Buxton, referred to in our last issue. These officers come into daily contact with a state of affairs in regard to our milk supply which makes them impatient of the more cautious attitude of the Central Departments. This impatience does them credit; they realise the vital part that the milk supply plays in the life of the nation; they feel that the law, or the way in which it is administered, is too considerate of the feelings of the farmer and the seller, with resultant damage to the health of the people. The following resolutions were passed :—

Milk supply : That in the opinion of this conference it is absolutely essential for the protection of infant life that definite standards be laid down by the Ministry of Health, below which the composition of genuine milk shall not fall. Further, that the necessary action to be taken, legal or otherwise, be decided by the Local Public Health Authorities.

That this conference views with great dissatisfaction the issue of the recent circular No. 325, by the Ministry of Health, relating to the institution of legal proceedings in respect of the sale of milk below the limits fixed by the regulations, and calls for its withdrawal in the interests of a pure milk supply.

The resolutions, especially the latter, are a good deal milder than some of the observations heard on the subject. As to circular 325, in which local authorities are instructed that no proceedings should be taken against vendors of milk which is deficient in fat without a series of tests (and not merely a single test) having been made, one speaker said that every local authority with whom he came into contact thought that the Ministry had gone "stark raving mad."

The case for the exercise of discretion in taking proceedings under the Sale of Milk Regulations was put with some force in the paper read by Mr. Hay Garth Brown, of the Ministry of Agriculture, on "the Administration of the Law relating to the Sale of Milk." Certain facts, said Mr. Brown at the outset, were common ground. There is no standard of quality for milk. The regulations merely provide that where milk contains less than certain percentages of milk fat and milk solids other than milk fat, it is to be assumed, until the contrary is proved, that it is not genuine. Again, the composition of milk varies within very wide limits. Experiments by trained observers have shown that variations in fat in the same herd of cows may be as wide as from 2 per cent. to 4.8 per cent.; that the percentage of fat is affected by the length of time elapsing between successive milkings; that milk secreted during the day is richer in fat than that secreted at night. Other causes of variation are age, intervals after calving, &c. On the other hand, poor feeding affects quantity rather than quality, and there is apparently no foundation for the idea that it is possible to water the milk through the cow's mouth.

Deficiencies in the composition of milk being so largely due to natural causes, and not to deliberate adulteration, the Ministry of Agriculture advocate the policy of enquiry and consideration of all the circumstances rather than the carrying of a case at once into the Police Court. How this policy can best be carried out, says Mr. Brown, depends on circumstances, but generally it means taking a series of informal samples, inquiries as to the circumstances in which the milk was forwarded, by the "appeal to the cow," and by taking a second formal sample. Mr. Brown did not think that any of those present would see legislation giving effect to a proposal for fixing a standard for milk and for making it an offence to sell milk below that standard, although he was in favour of every inducement being offered to purveyors to supply milk which was not only not adulterated but contained a high percentage of butter fat, so that the selling of milk would become an unprofitable occupation.

Mr. J. T. Quinton, of Liverpool, speaking from long experience, and regarded by the sanitary inspectors themselves as especially well able to handle his subject, spoke at Buxton in favour of a standard below which the quality of milk should not be allowed to fall. He reviewed certain legal decisions, including the outstanding case of Hunt *v.* Richardson, where it was established that, in the present state of the law, milk poor in value, deficient in fat, coming from unhealthy cows, could be sold without penalty, because it could be shown to have come direct from the cow and to have been strained for impurities. Mr. Quinton was able to quote Lord Reading in favour of the view that such a state of the law is unsatisfactory, he having, in the course of a judgment, stated that the arguments had convinced him as to the need for a reconsideration by the authorities of the whole question with a view to legislation.

In regard to a minimum limit for the composition of milk, Mr. Quinton agrees that such a limit must in every case be tested by reference to the average of a herd, and considers that this affords ample protection to the farmer. He is definitely not in favour of the official proposals for the grading of milk; a view in which he was supported in the subsequent discussion by Alderman Muirhead, the chairman of the Liverpool Health Committee, who said that housewives would buy poor grade milk because it was cheaper, just as they will buy flannelette instead of flannel.

The speaker urged the need for research in regard to breed, rations, environment. He condemned the section (s. 9) of the Milk and Dairies Act—which protects the seller of milk against prosecution if churns are sealed when they leave him and not so sealed when arriving at their destination—as retrograde and administratively impracticable ; a statement which found a resounding echo from the sanitary inspectors present.

The factors in producing pure, clean milk are so simple that the sanitary inspector is moved to indignation, and impatient of the care bestowed on the farmer. They are : Simple buildings, well-lit and well-ventilated ; clean cows ; clean utensils ; the personal care and interest of the proprietor.

In the subsequent discussion, the following points were made :—

The pasturage is too often impoverished.

The water supply is frequently "catch-as-catch-can."

The housing campaign should start with the cow sheds.

Better milk comes from the city cowsheds than from outside.

A standard is always better than presumptive limits, but will be better attained by education and enlightenment rather than by wholesale prosecutions.

Clean milk will mean better business.

Healthy competition has been promoted in one county with prizes shared by the cowmen.

The whole discussion, following a first-rate paper, was on a good level, and is deserving the attention of those in authority, as coming from first-hand observation of the " eyes, ears and noses " of the Health Authorities.

